# Author Correction: Dual-site segmentally synergistic catalysis mechanism: boosting CoFeS_x_ nanocluster for sustainable water oxidation

**DOI:** 10.1038/s41467-024-52015-z

**Published:** 2024-10-07

**Authors:** Siran Xu, Sihua Feng, Yue Yu, Dongping Xue, Mengli Liu, Chao Wang, Kaiyue Zhao, Bingjun Xu, Jia-Nan Zhang

**Affiliations:** 1 Key Laboratory of Advanced Energy Catalytic and Functional Materials Preparation, College of Materials Science and Engineering, Zhengzhou University, Zhengzhou, 450001 China; 2 National Synchrotron Radiation Laboratory, University of Science and Technology of China, Hefei, 230026 Anhui China; 3 College of Chemistry and Molecular Engineering, Peking University, Beijing, China; 4 State Key Laboratory of Coking Coal Resources Green Exploitation, Zhengzhou, 450001 China

**Keywords:** Electrocatalysis, Catalytic mechanisms, Heterogeneous catalysis

Correction to: *Nature Communications* 10.1038/s41467-024-45700-6, published online 26 February 2024

The original version of Figure 4 of this Article contained some minor errors in the drawing of the schematic diagram of Figure 4e. Figure 4 has been updated in both the PDF and HTML versions of the Article.

