# Supplementary material for: Dual-site segmentally synergistic catalysis mechanism: boosting CoFeSx nanocluster for sustainable water oxidation
Source: Nat Commun. 2024 Feb 26;15:1720. doi: 10.1038/s41467-024-45700-6 (PMC10897303; doi:10.1038/s41467-024-45700-6)
Supplement: Supplementary file 1 — Supplementary Information [file 41467_2024_45700_MOESM1_ESM.pdf]

Supplementary Materials for

Dual-site segmentally synergistically catalysis mechanism: boosting $CoFeS_x$ nanocluster for sustainable water oxidation

Siran Xu *et al.*

*Corresponding author. Email: *zjn@zzu.edu.cn*

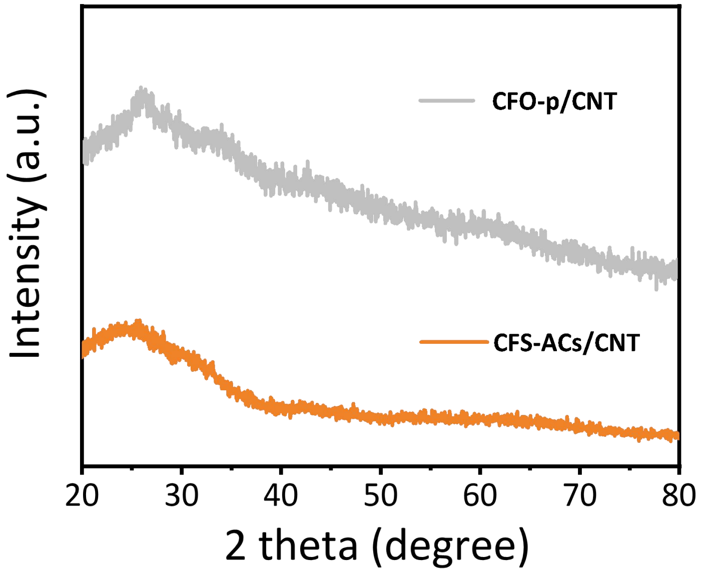

Supplementary Fig. S1 XRD patterns of CFO-p/CNT and CFS-ACs/CNT.

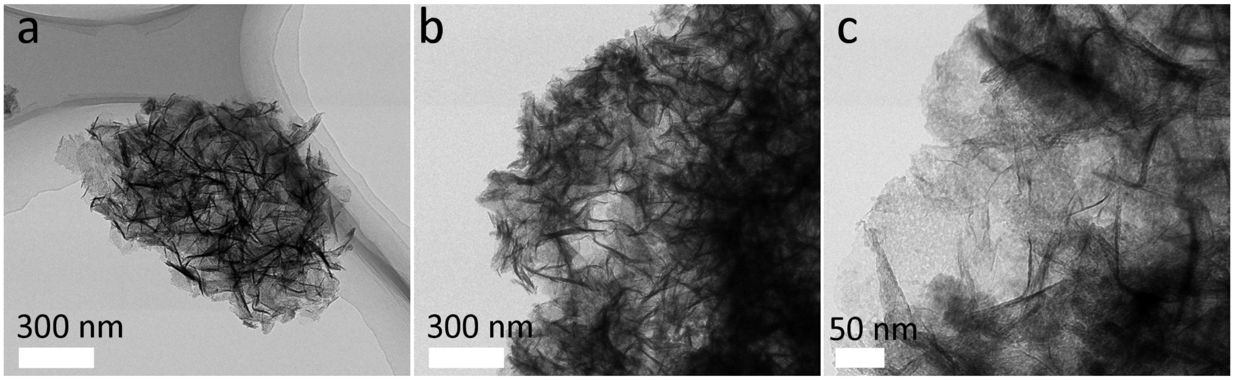

Supplementary Fig. S2 TEM images of the CoFeO*x* nanosheet with different scales of (a,b) 300 nm and (c) 50 nm.

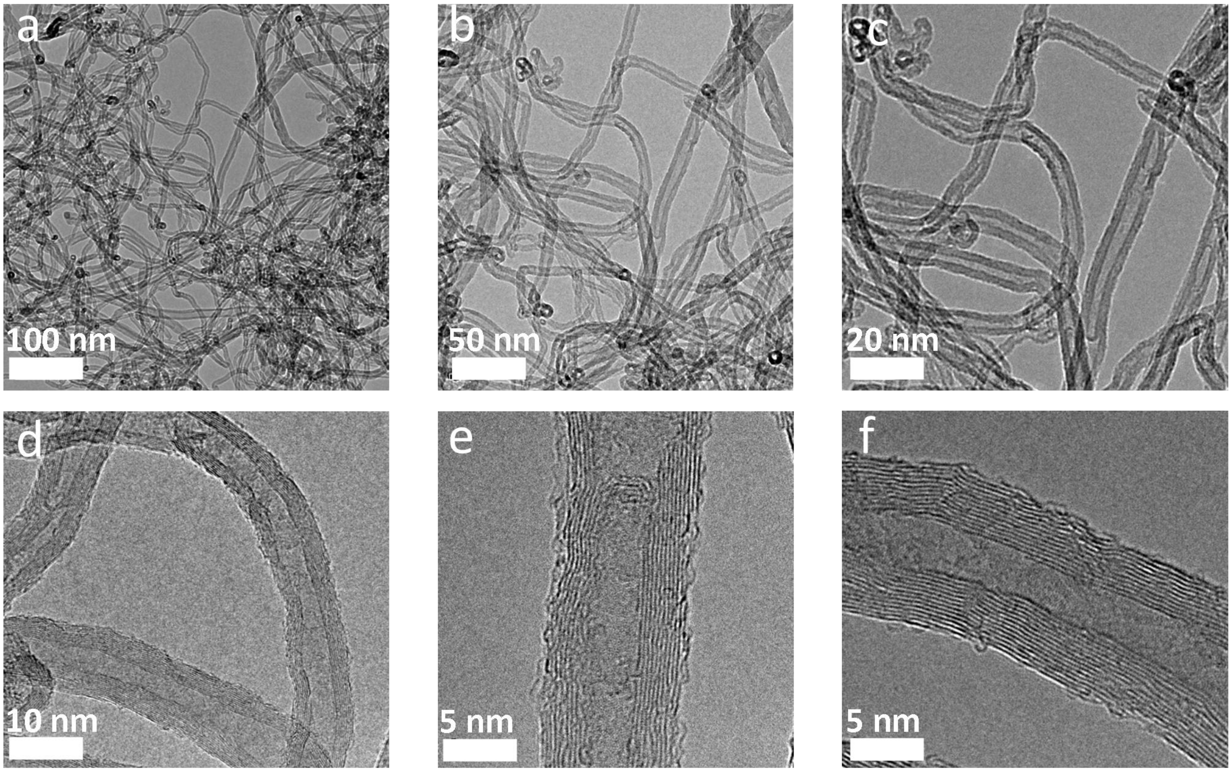

Supplementary Fig. S3 TEM images of MWCNT with different scales of (a) 100 nm, (b) 50 nm, (c) 20 nm, (d) 10 nm and (e,f) 5 nm.

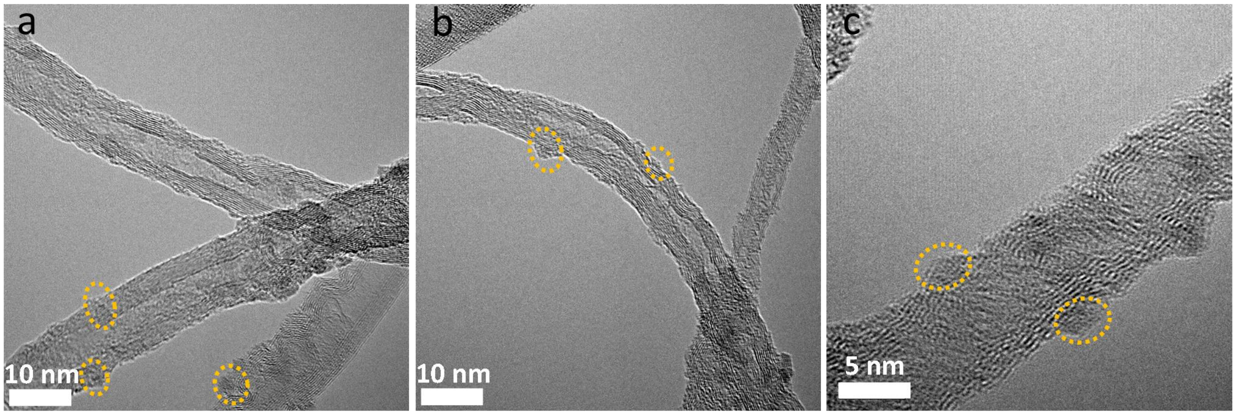

Supplementary Fig. S4 TEM images of the CFO-p/CNT (yellow circle is CFO-p nanosheets precursor) with different scales of (a,b) 10 nm and (c) 5 nm.

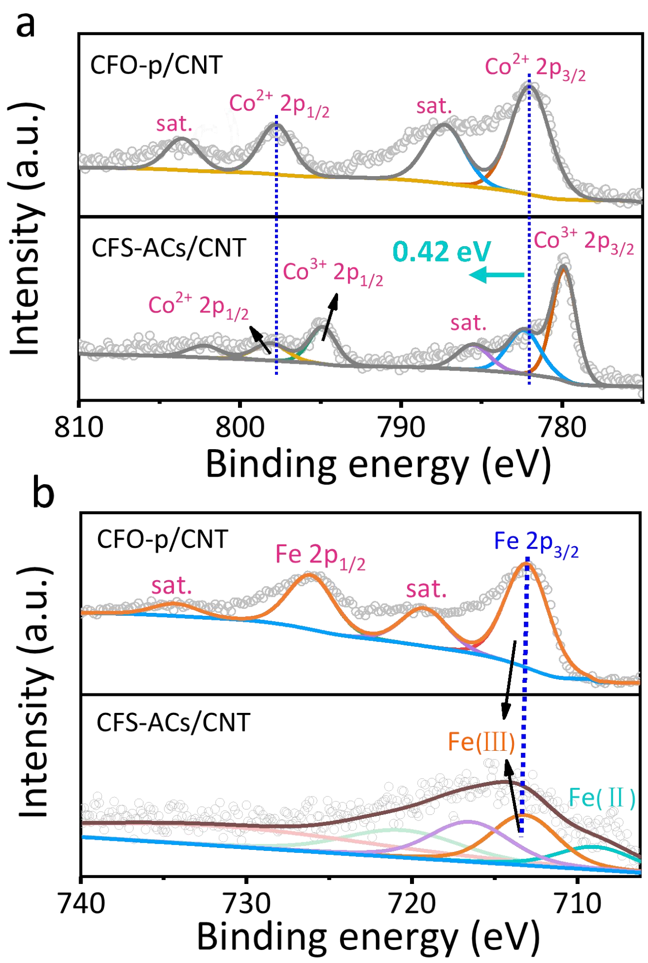

Supplementary Fig. S5 XPS spectra. Co 2p (a) and Fe 2p (b) of CFO-p/CNT and CFS-ACs/CNT, respectively.

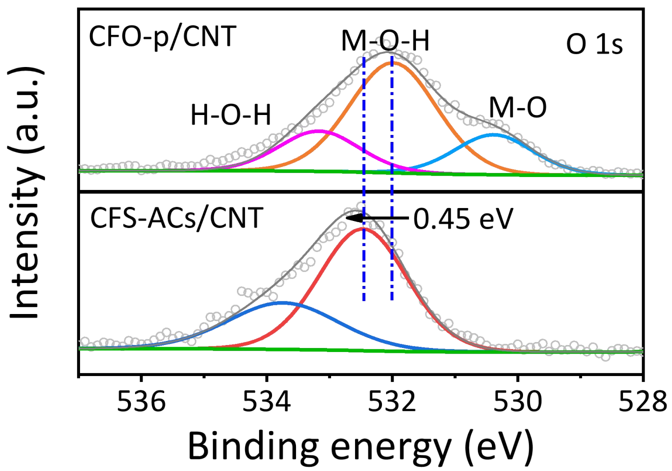

Supplementary Fig. S6 XPS spectra of O 1s of CFO-p/CNT and CFS-ACs/CNT, respectively. O1s spectrum of CFO-p/CNT can observe three peaks at 530.36 eV (Fe (Co)-O), 531.98 eV (Fe (Co)-OH) and 533.22 eV (H-O-H).

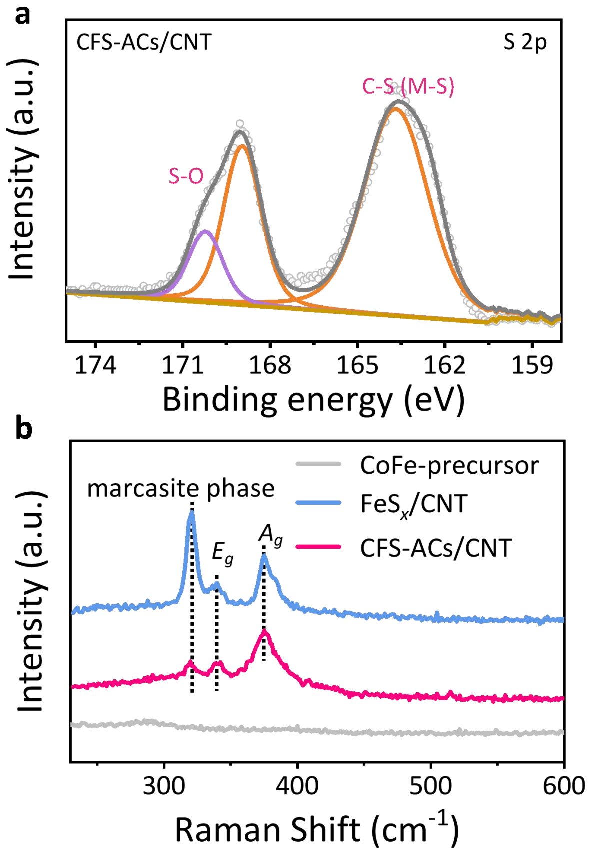

Supplementary Fig. S7 Structure analysis. (a) XPS spectra of S 2p of CFS-ACs/CNT, (b) Raman spectrum of CFS-ACs/CNT, FeS_x/CNT and CFO-p/CNT.

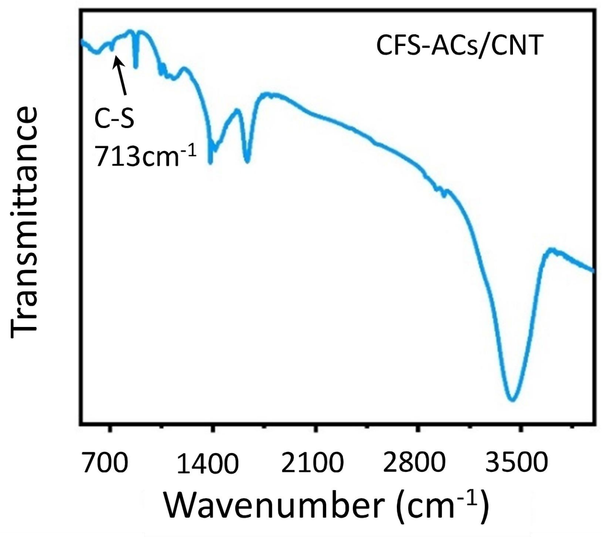

Supplementary Fig. S8 FT-IR spectrum of CFS-ACs/CNT.

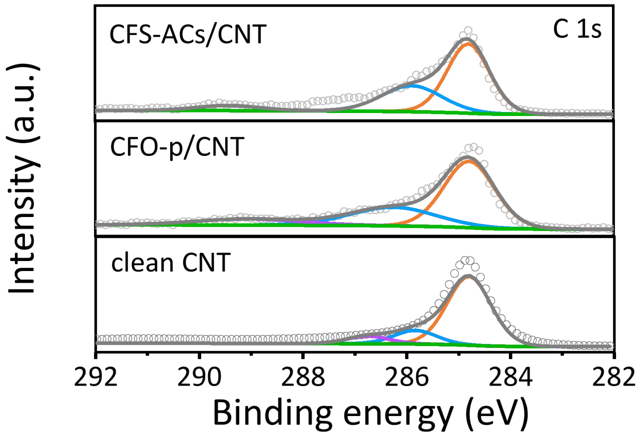

Supplementary Fig. S9 XPS spectra for C 1s of CNT, CFO-p/CNT and CFS-ACs/CNT.

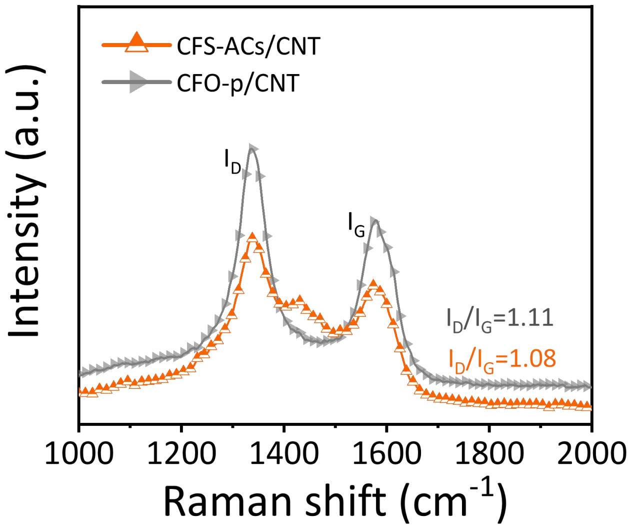

Supplementary Fig. S10 Raman spectrum of CFO-p/CNT and CFS-ACs/CNT.

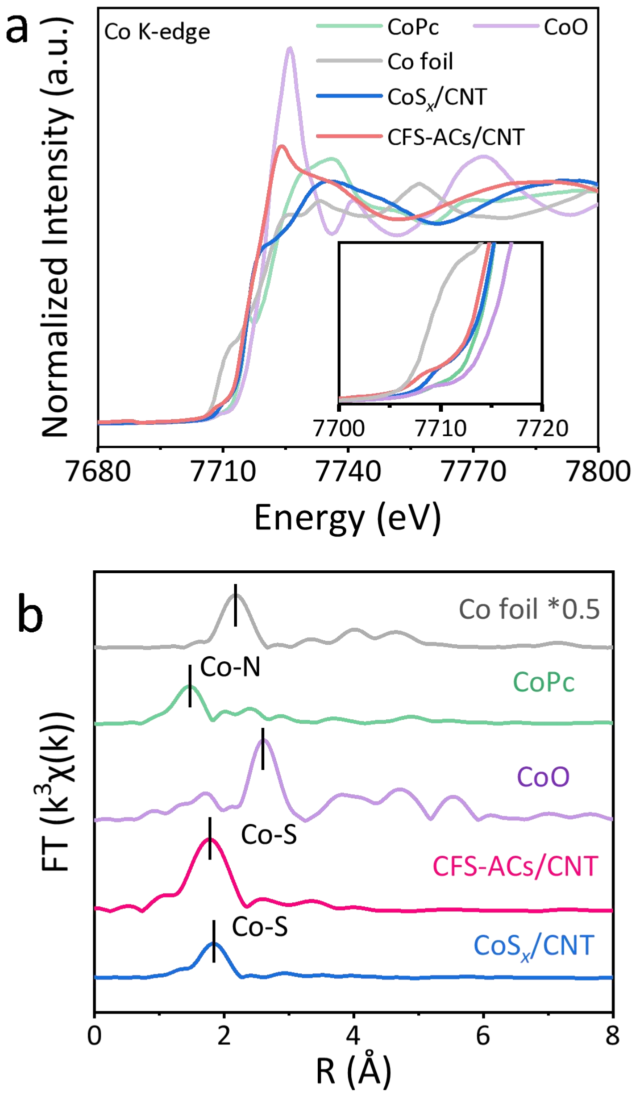

Supplementary Fig. S11 XAS analysis. (a) Co K-edge XANES and (b) Fourier-transform EXAFS spectra for CFS-ACs/CNT and CFO-p/CNT.

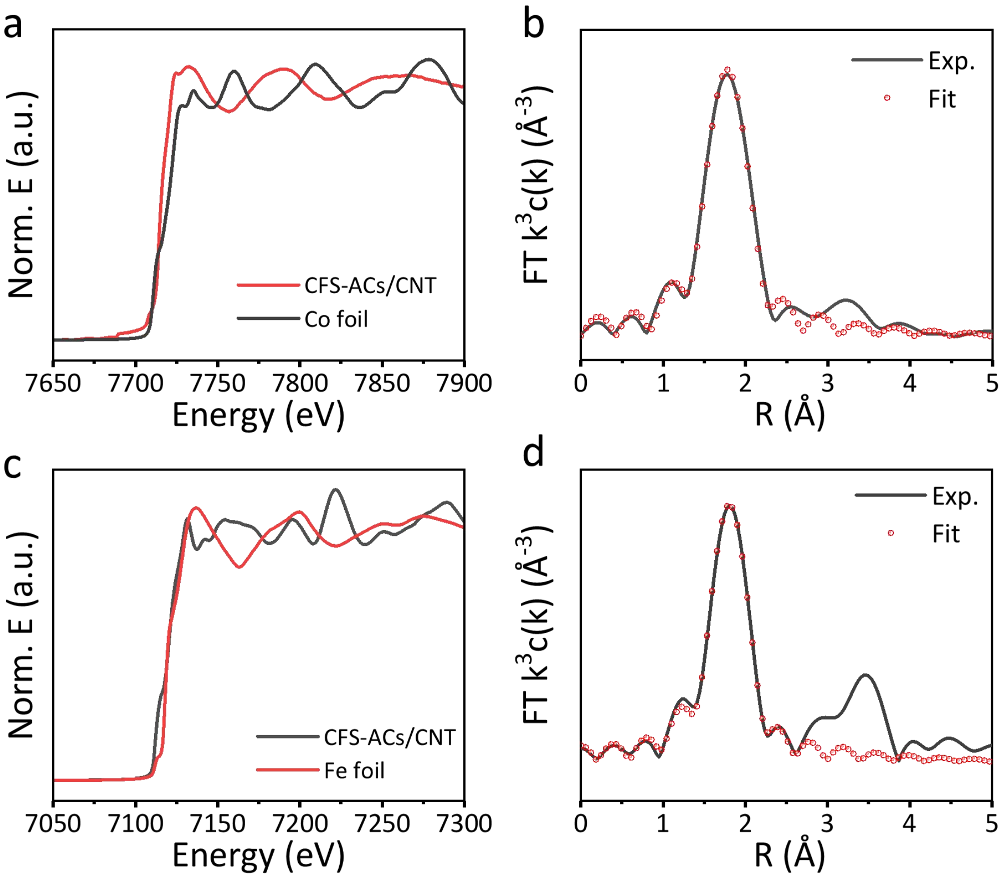

Supplementary Fig. S12 EXAFS fitting curves. CFS-ACs/CNT sample in *R* space of Co (a,b) and Fe (c,d).

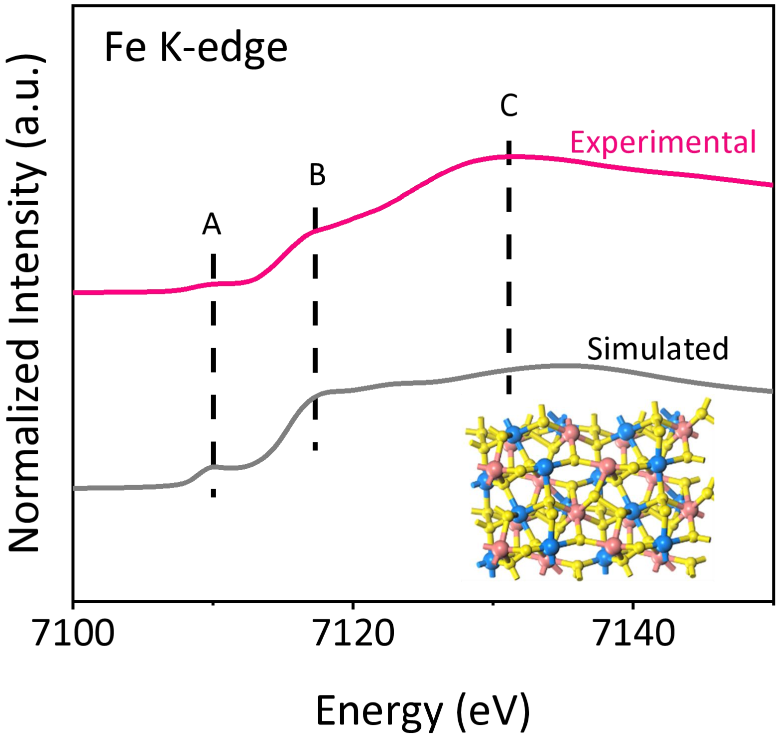

Supplementary Fig. S13 Comparison between the experimental Fe K-edge XANES spectra of CFS-ACs/CNT catalyst and the theoretical spectra calculated based on $CoFeS_4$ structure embedded in the carbon matrix.

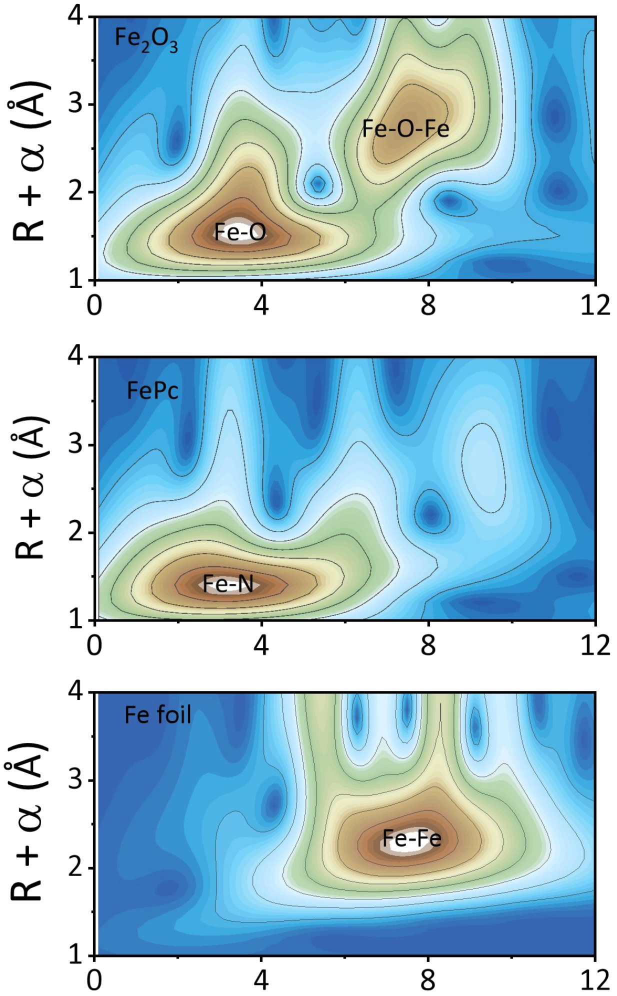

Supplementary Fig. S14 Wavelet transforms of the experimental k^3-weighted EXAFS spectra of references of Fe foil, FePc and Fe_2O_3.

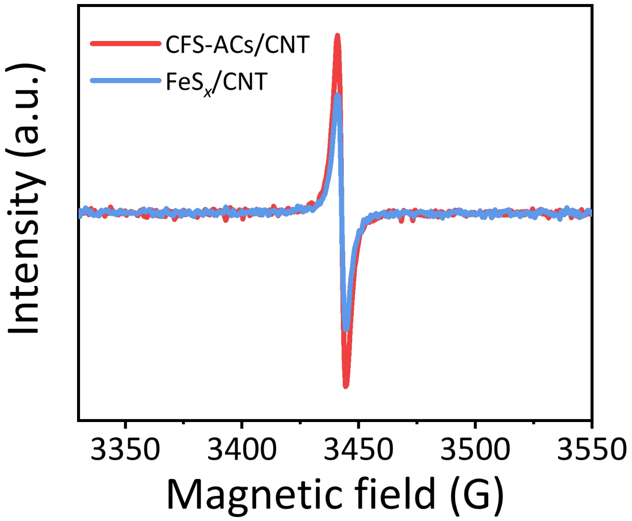

Supplementary Fig. S15 EPR spectra with FeS_x/CNT and CFS-ACs/CNT.

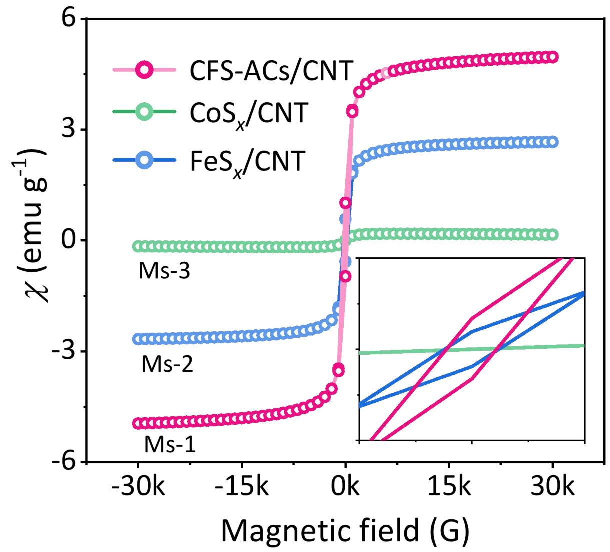

Supplementary Fig. S16 Magnetic hysteresis loops of CFS-ACs/CNT, FeS$_x$/CNT and CoS$_x$/CNT at room temperature (300 K).

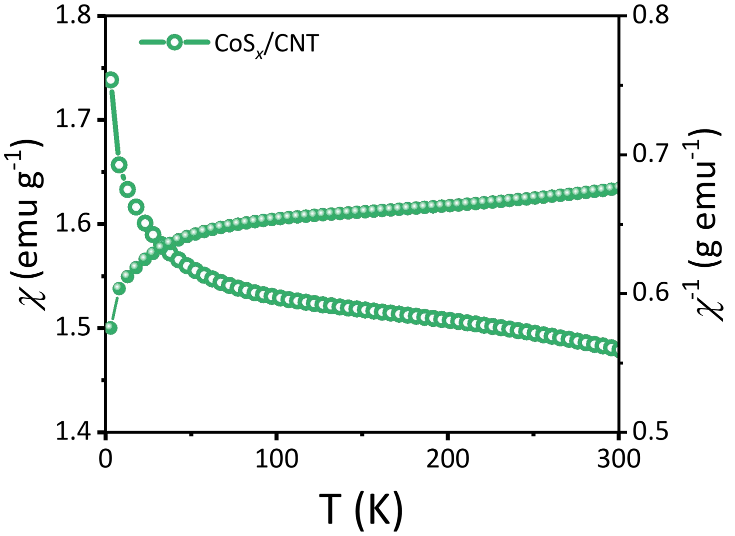

Supplementary Fig. S17 M–T susceptibility χ and reciprocal 1/ χ of CoS$_x$/CNT.

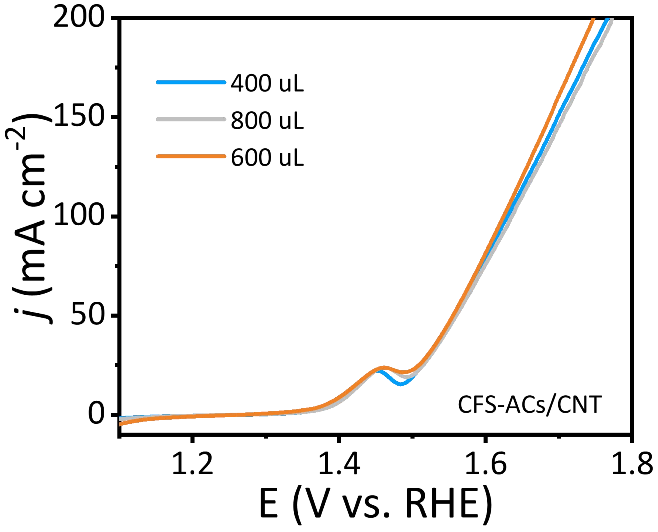

Supplementary Fig. S18 LSV curves of different content of CFS-ACs/CNT ink on NF substrate.

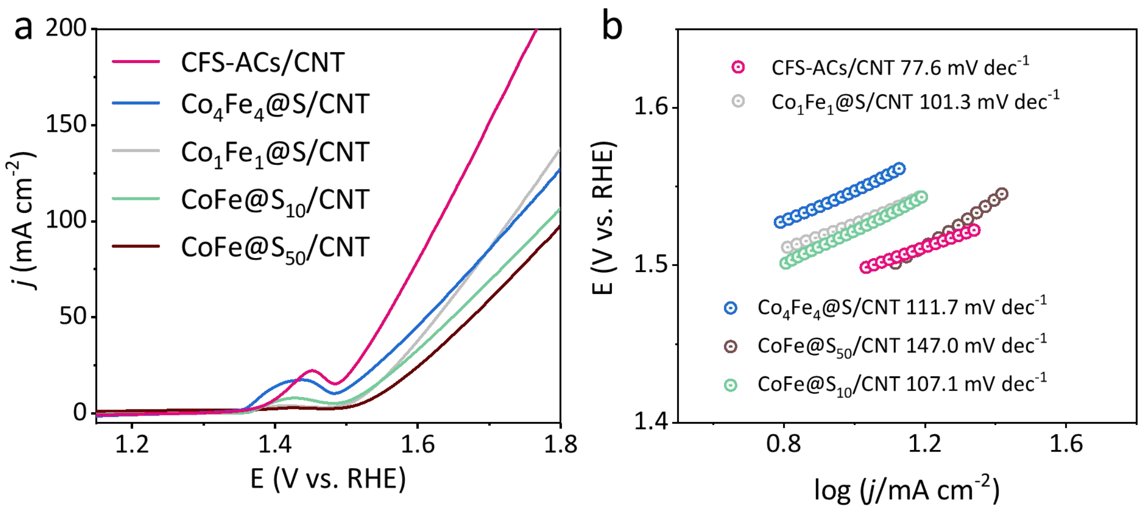

Supplementary Fig. S19 OER performance of as-prepared catalysts with different atom ratios. (a) LSV curves, (b) Tafel slopes of different content of CFS-ACs/CNT.

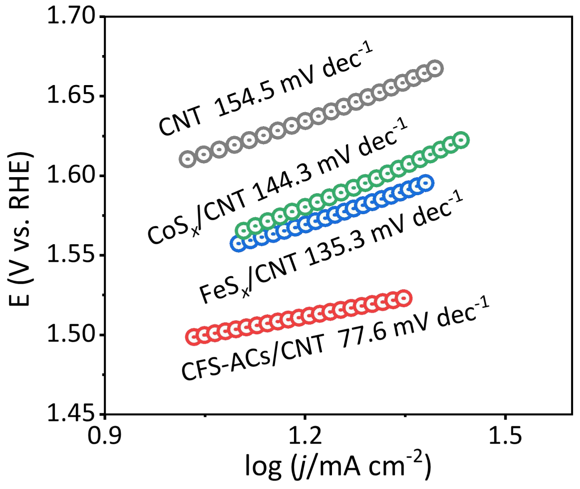

Supplementary Fig. S20 Tafel slope of CFS-ACs/CNT, FeS$_x$/CNT, CoS$_x$/CNT and pure CNT.

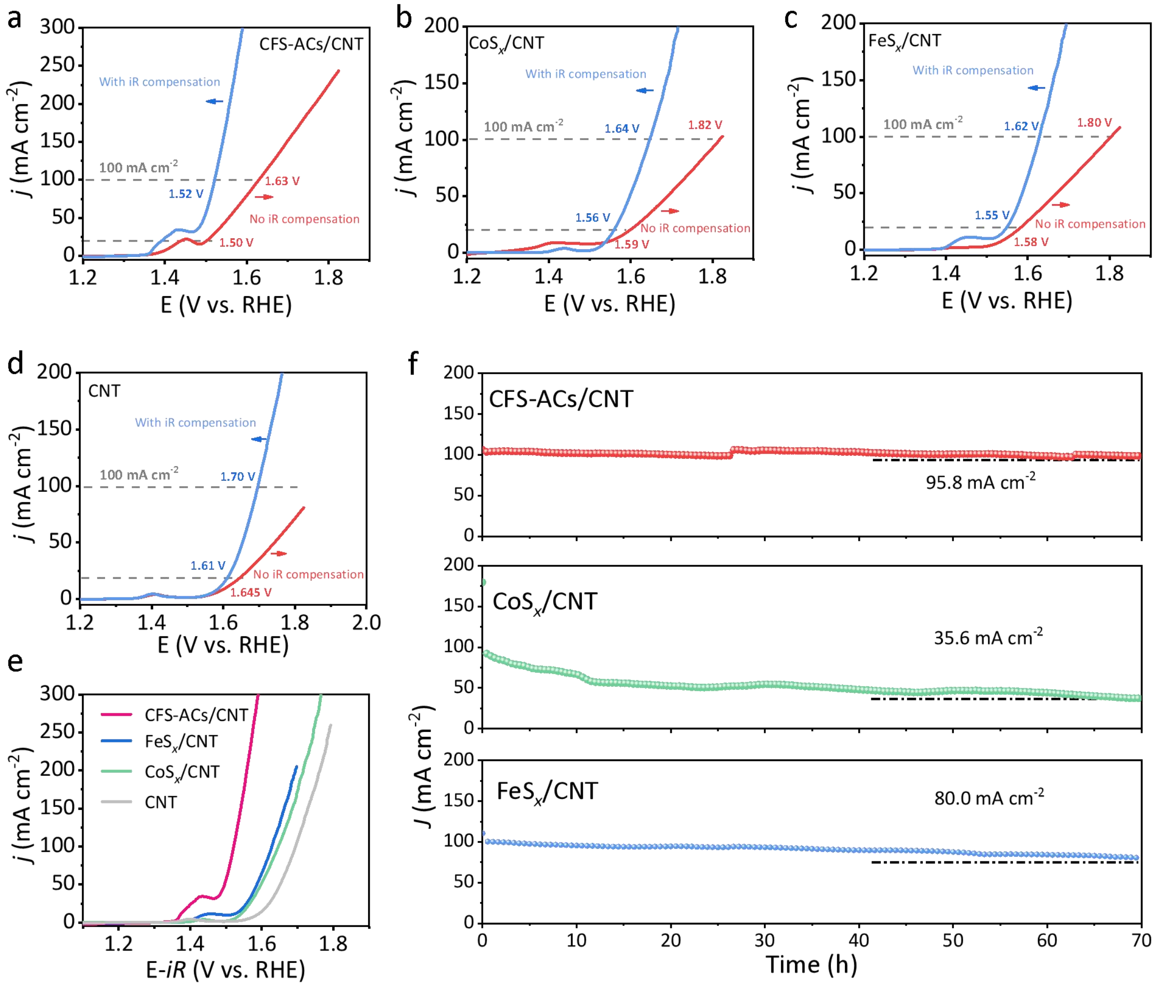

Supplementary Fig. S21 OER performance before and after *iR* compensation. (a-d) LSV curves before and after *iR* compensation of CFS-ACs/CNT, FeS_x/CNT, CoS_x/CNT and pure CNT. (e) LSV curves of CFS-ACs/CNT, FeS_x/CNT, CoS_x/CNT and pure CNT with 80% *iR* compensation. (f) The chronoamperometry curve of CFS-ACs/CNT, FeS_x/CNT and CoS_x/CNT at 100 mA cm^{-2} with 80% *iR* compensation.

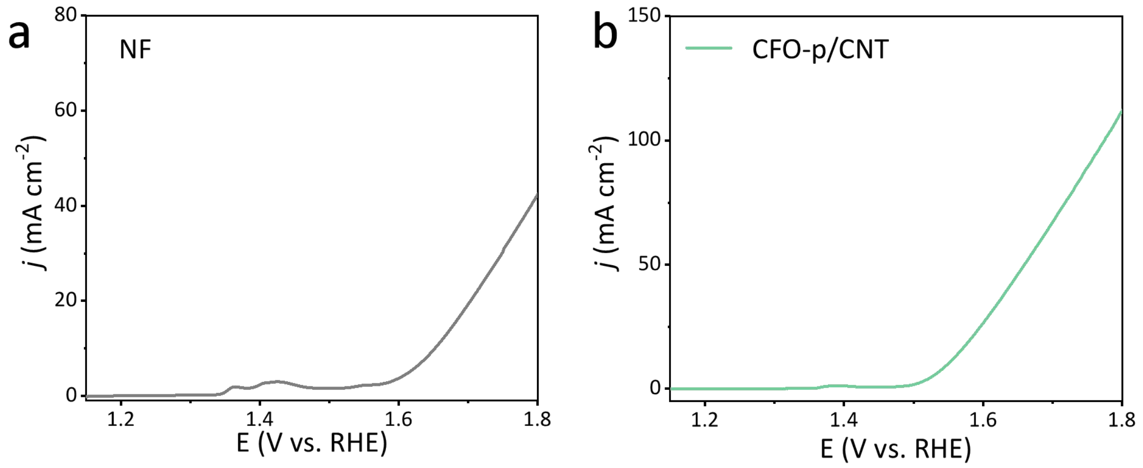

Supplementary Fig. S22 LSV curves of (a) pure NF substrate, (b) CFO-p/CNT.

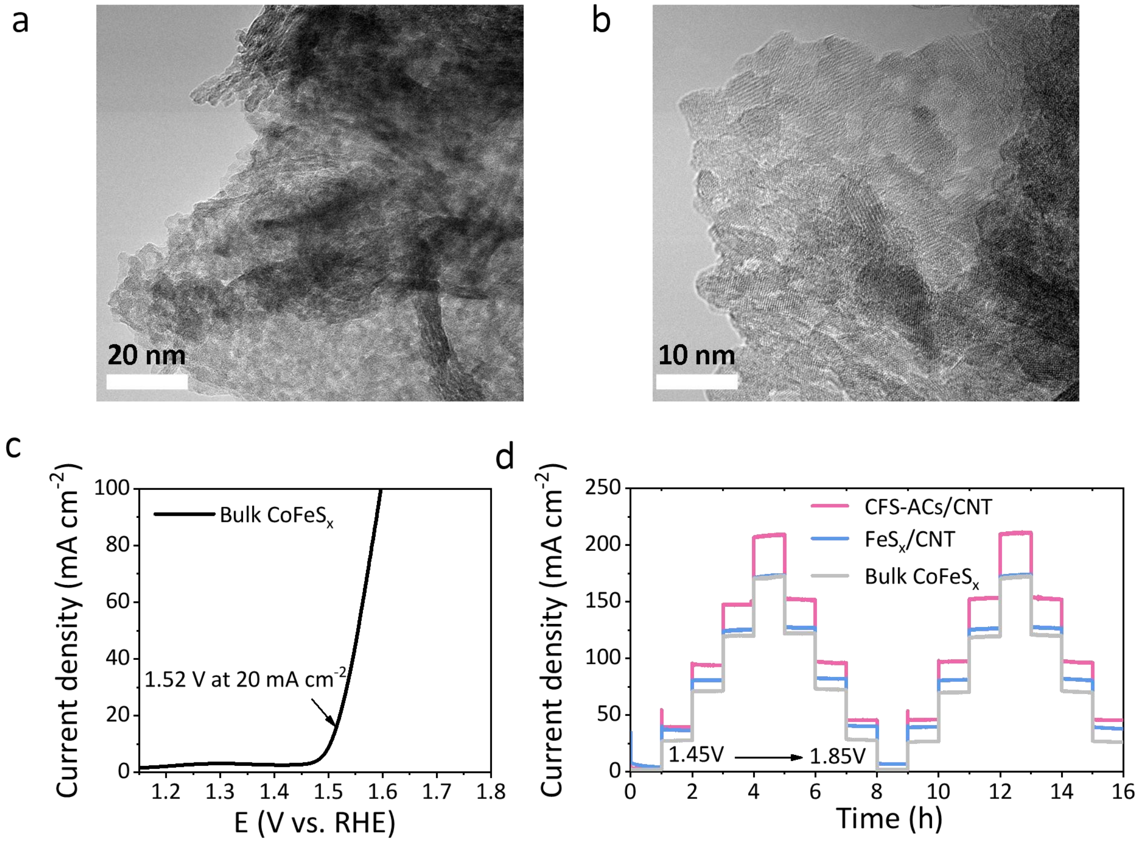

Supplementary Fig. S23 OER performance evaluation of bulk CoFeS$_x$. (a,b) TEM images of bulk CoFeS$_x$, (c) LSV curve, (d) current density plot at different potential from 1.45 V to 1.85 V *vs.* RHE among CFS-ACs/CNT, FeS$_x$/CNT and bulk CoFeS$_x$.

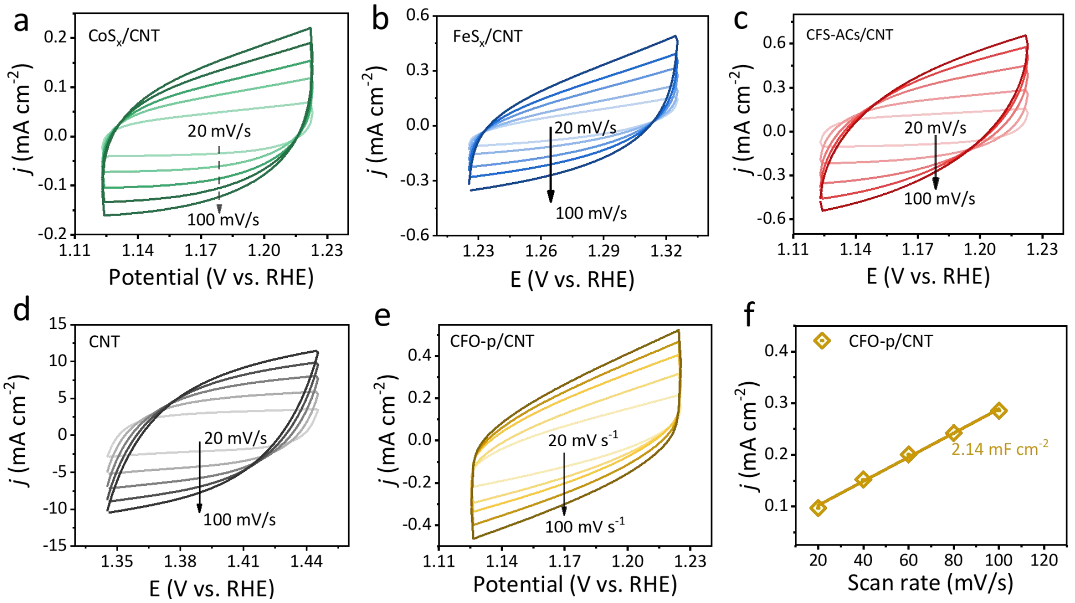

Supplementary Fig. S24 CV curves with different rate from 20 to 100 mV s^{-1}. (a) CoS_x/CNT, (b) FeS_x/CNT, (c) CFS-ACs/CNT, (d) CNT, (e) CFO-p/CNT and (f) C_{dl} curves of CFO-p/CNT.

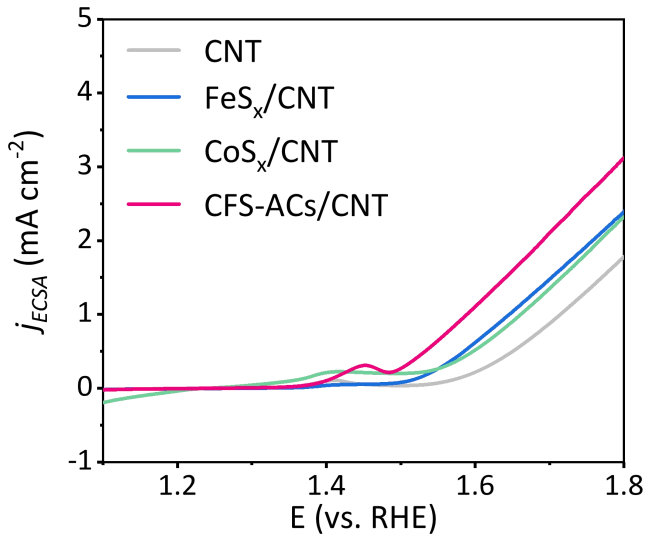

Supplementary Fig. S25 ECSA calibrated LSV curves of CFS-ACs/CNT, FeS_x/CNT, CoS_x/CNT, pure CNT, commercial IrO_2 and Ni foam substrate.

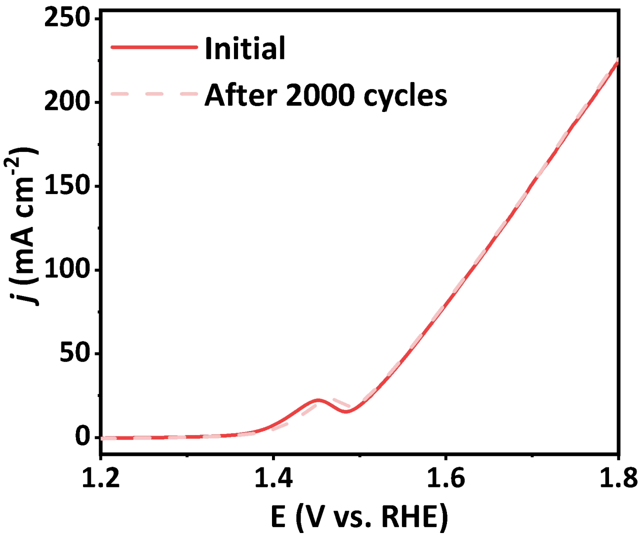

Supplementary Fig. S26 LSV curves of CFS-ACs/CNT before and after 2000 CV cycles.

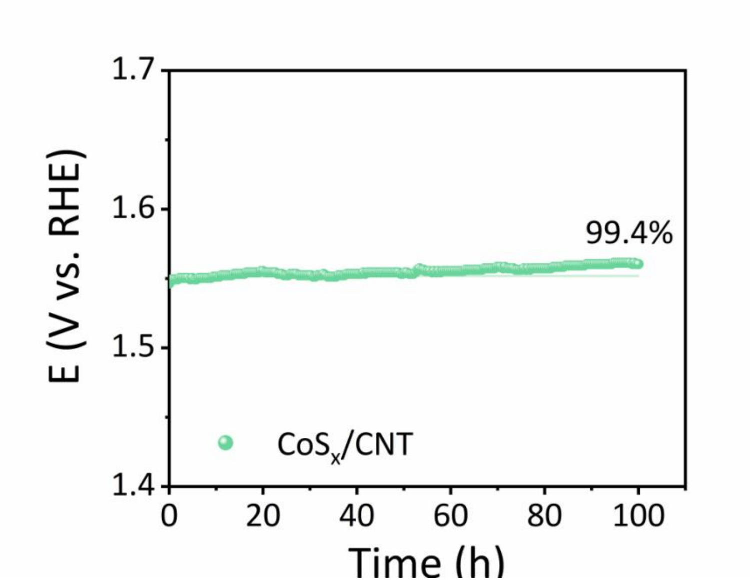

Supplementary Fig. S27 The chronopotentiometry curve of CoS_x/CNT in electrolyte with 1.0 M KOH.

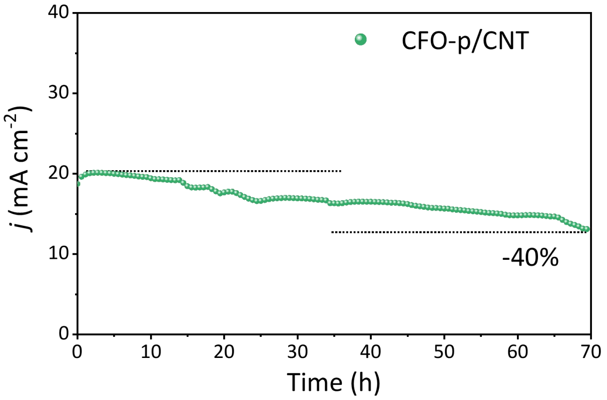

Supplementary Fig. S28 The chronopotentiometry curve of CFO-p/CNT in electrolyte with 1.0 M KOH.

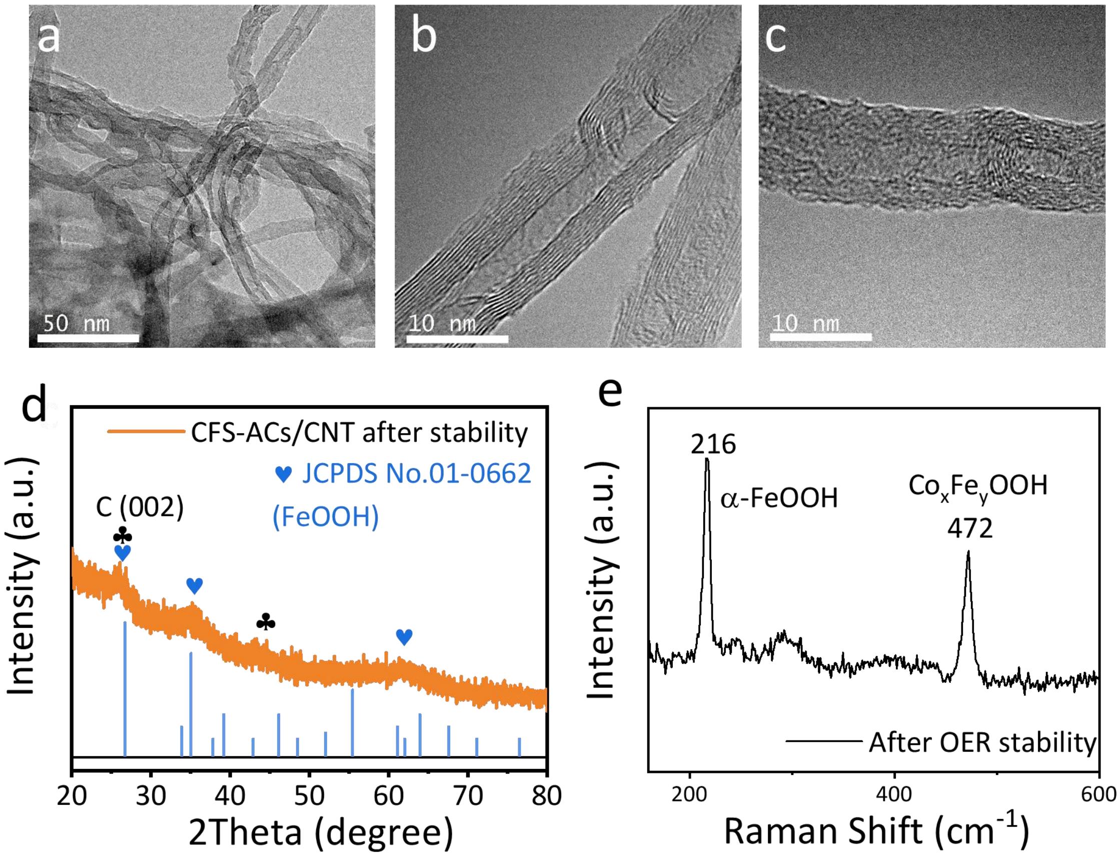

Supplementary Fig. S29 Structural and OER performance characterization. (a-c) TEM images, (d) XRD pattern, and (e) Raman spectra of CFS-ACs/CNT after long-term stability of about 633 h.

Supplementary Note 1 As shown in Figure S29a-c, TEM images after OER stability of CFS-ACs/CNT did not show significant particles produced by agglomeration of clusters. As shown in Figure S29d, XRD pattern after OER stability showed that three weak diffraction peaks belonging to Co(Fe)-OOH, and two weak peaks located at 26.6° (002) and 43.0° (100) belongs to carbon nanotube. The metastable structure determines its weak crystallinity, so it is difficult to obtain obvious the specific crystal phase structure and exposed crystal faces[1], the clusters do not agglomerate into a highly crystalline phase after stabilization, thus XRD only obtained weak characteristic peaks of FeOOH and graphitic carbon. As shown in Figure S29e, Raman spectroscopy after OER stability show that the main peak at 216 and 472 cm^{-1} are attributed to E_g and A_g modes of Fe-O and Co_xFe_y-O bonds, respectively[2, 3]. The existence of CoFeOOH phase show that the partially oxidized on the surface of $CoFeS_x$, which is matched with XPS spectra.

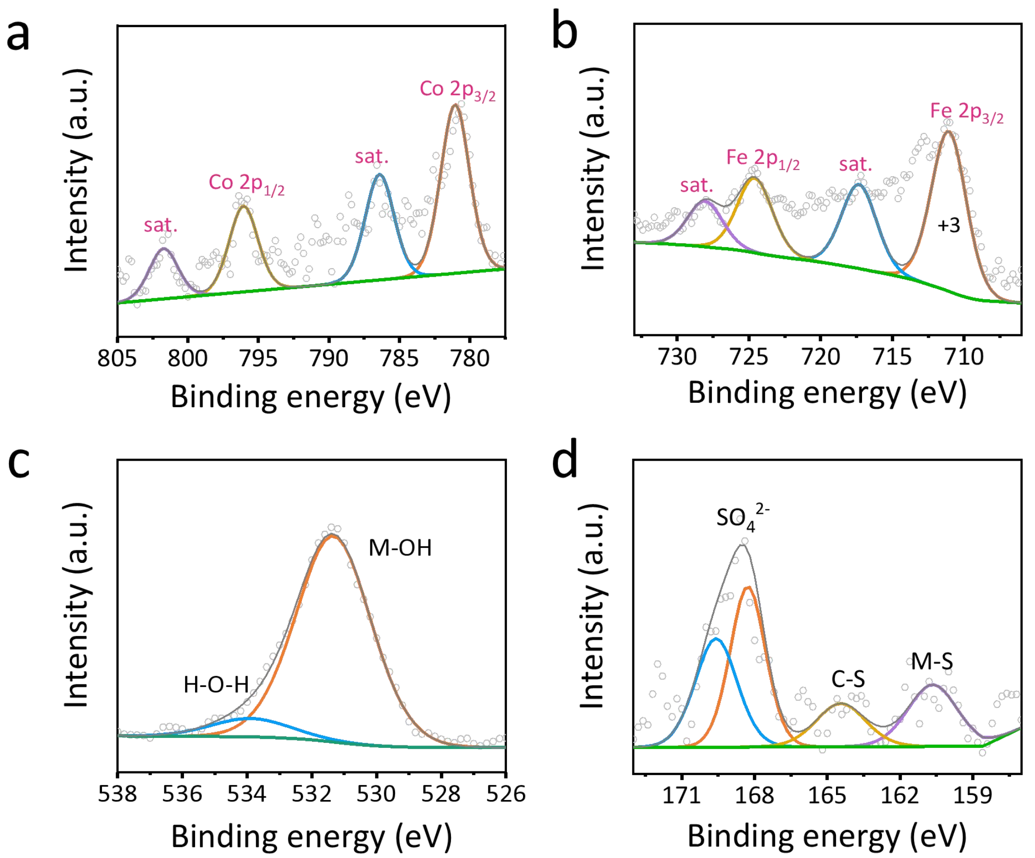

Supplementary Fig. S30 XPS spectra. (a) Co 2p, (b) Fe 2p, (c) S 1s and (d) O 1s of CFS-ACs/CNT after long-term stability of about 633 h.

Supplementary Note 2 The enhanced signal of the M-OH peak in the O 1s spectrum of CFS-ACs/CNT during the OER process indicates that an increase in M-OH bonding after the surface S-induced Co-Fe compounds are used for enhanced oxygen precipitation reaction reconstitution, which implies the generation of surface metal (oxygen) hydroxides. After OER process of 633 h, the feature peak belongs to C-S-C decreases obviously, which further implying that the excellent long-term stability is related to the content of C-S-C. Meanwhile, and a peak of SO_4^{2-} corresponding to S oxidation appears at 168.5 eV is enhanced, which indicates leaching and oxidation of S elements during the OER process with SO_4^{2-} being adsorbed on the catalyst surface.

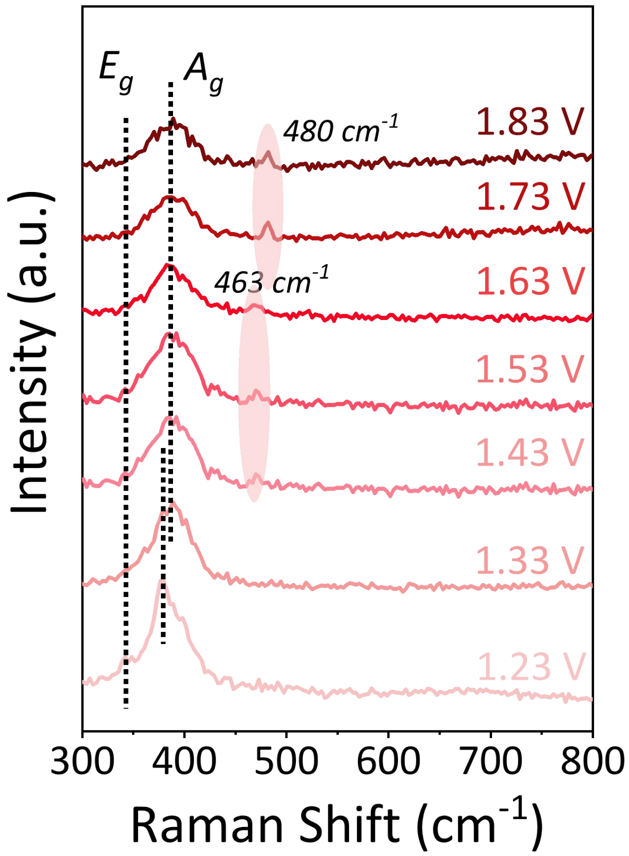

Supplementary Fig. S31 ***In-situ*** **Raman spectroscopy of CFS-ACs/CNT catalyst in 1.0 M KOH**.

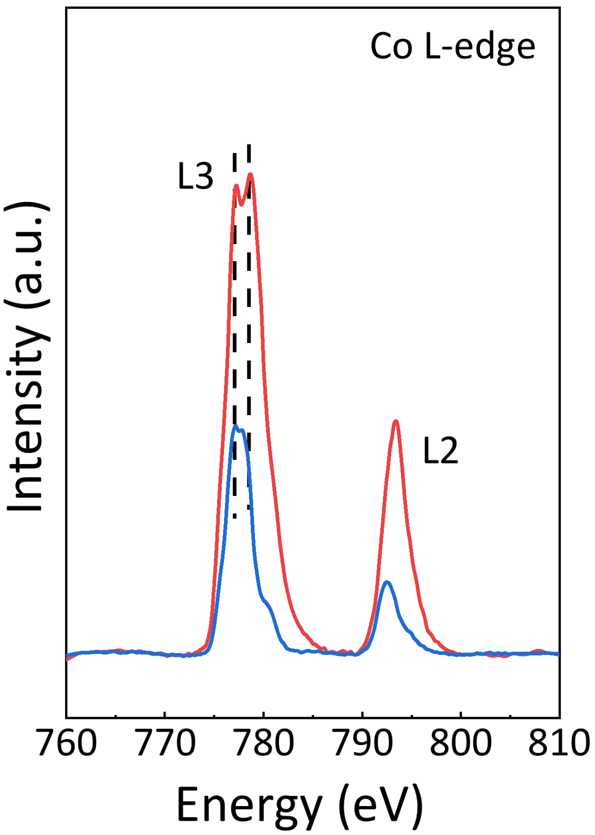

Supplementary Fig. S32 Co L-edge XANES spectra before and after stability of CFS-ACs/CNT.

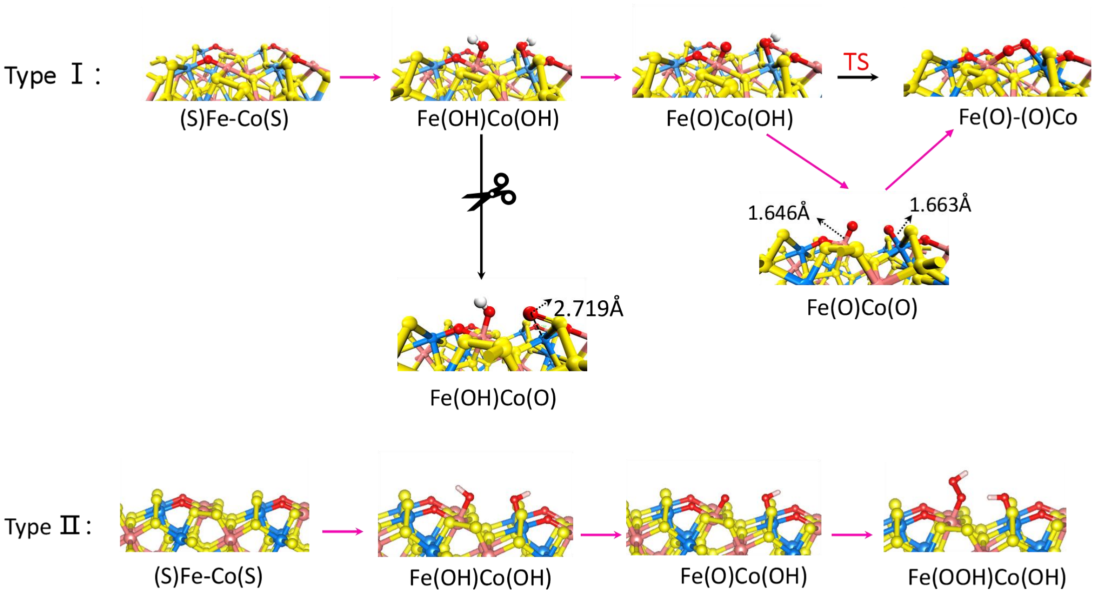

Supplementary Fig. S33 Calculation of the adsorption structures of different oxygen species on CFS. Two structural models corresponding to each specific oxygen species on CFS, respectively. In order to simulate the environment where oxygen is enriched on the electrode surface in actual electrocatalytic process, both Co and Fe atoms have been highly coordinated by O atoms.

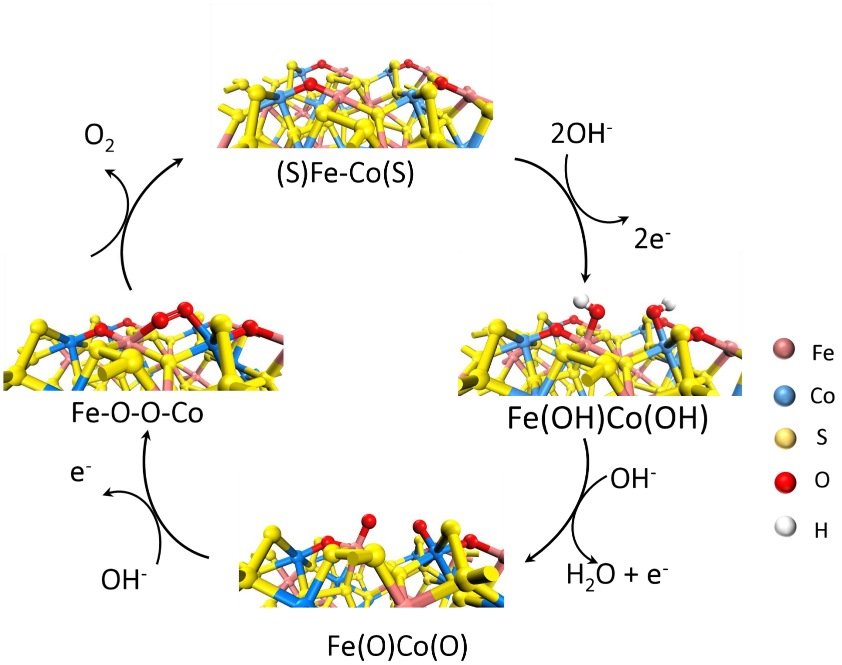

Supplementary Fig. S34 Proposed OPM mechanism of CoFeS$_x$/CNT. Simulate the 4e$^-$ mechanism of oxygen evolution reaction on CoFeS$_x$/CNT by the spin-polarized density functional theory.

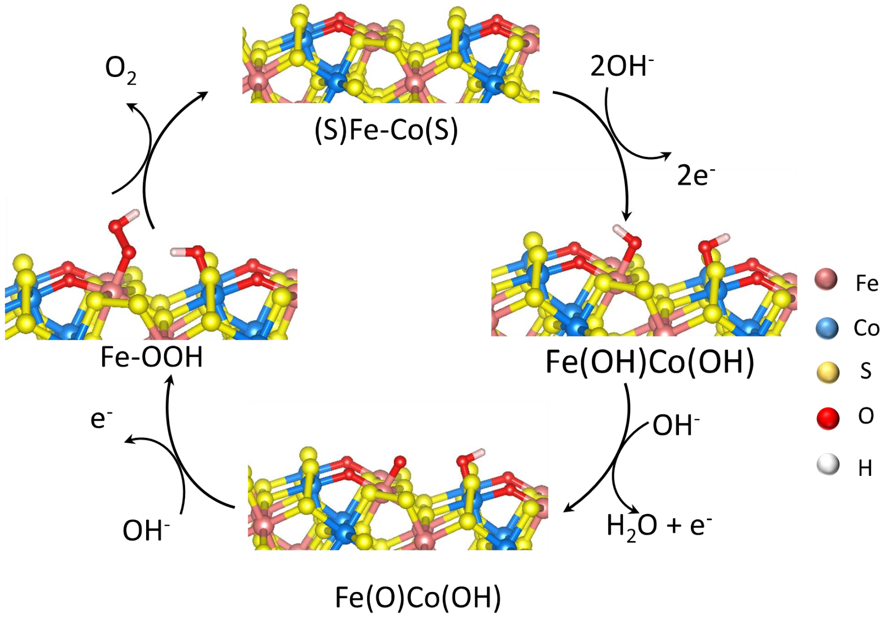

Supplementary Fig. S35 Typical AEM mechanism of dual-site catalyst. Simulate the 4e$^-$ mechanism of oxygen evolution reaction on $CoFeS_x$/CNT by the spin-polarized density functional theory.

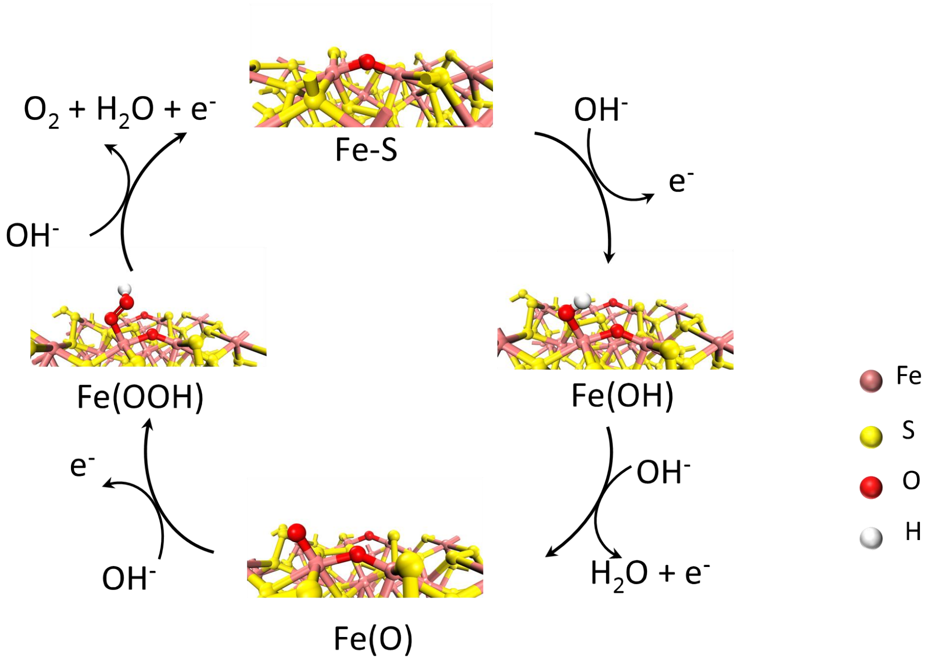

Supplementary Fig. S36 Proposed AEM mechanism of FeS_x/CNT. Simulate the $4e^-$ mechanism of oxygen evolution reaction on FeS_x/CNT by the spin-polarized density functional theory.

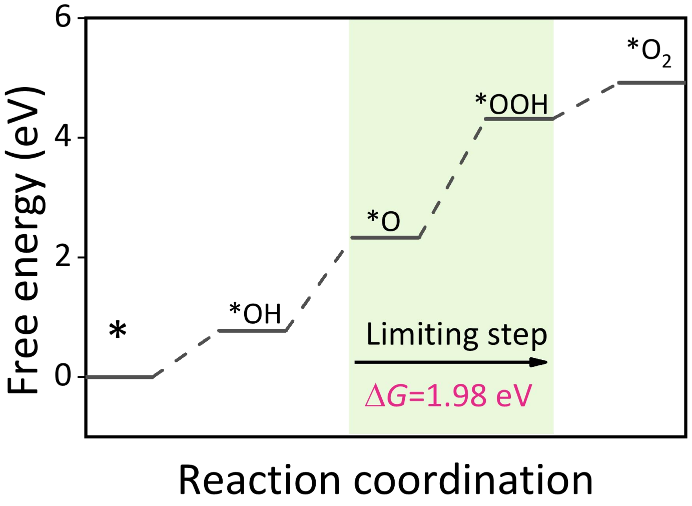

Supplementary Fig. S37 The free energy diagram (Δ*G*) of typical AEM mechanism of FeS_2 phase.

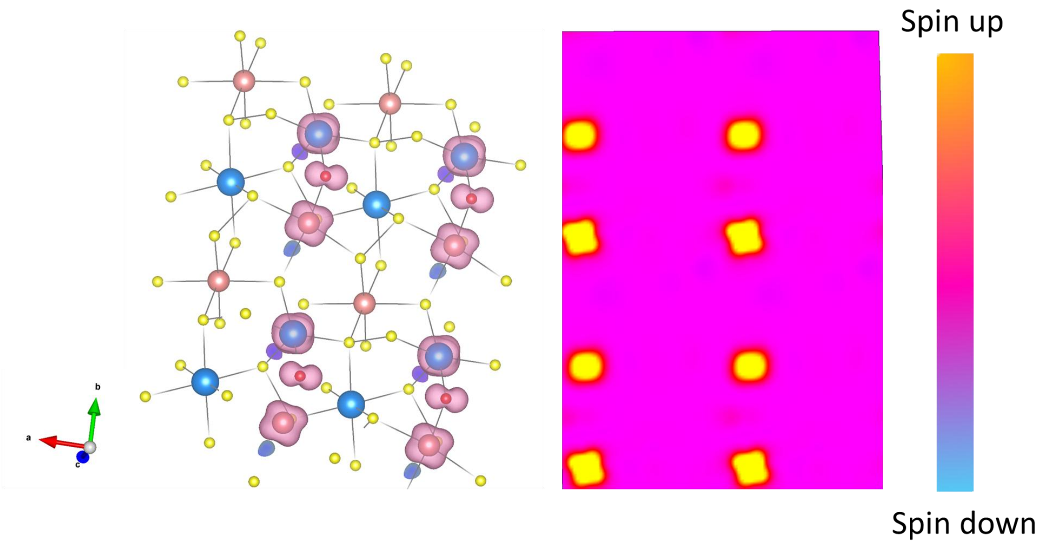

Supplementary Fig. S38 Simulated spin density map and planar distribution maps of the spin polarization of $CoFeS_4$ phase.

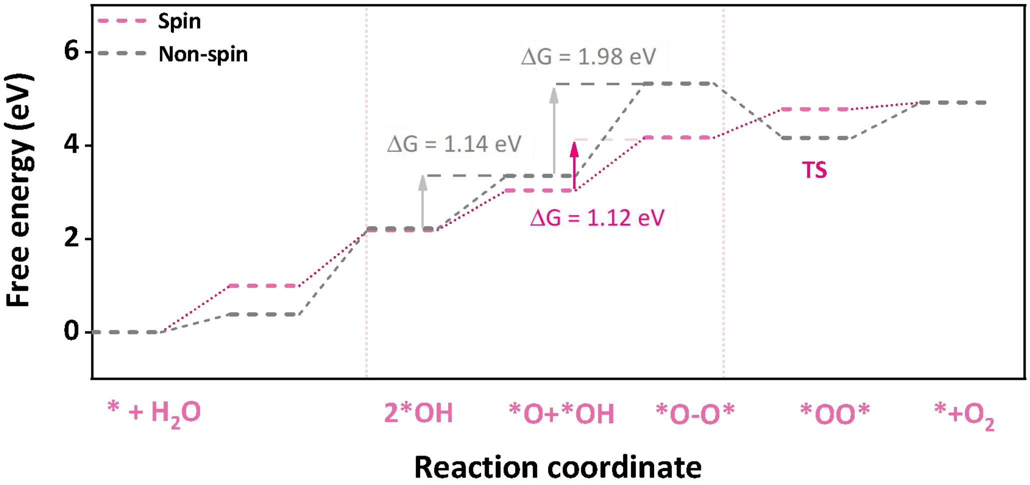

Supplementary Fig. S39 Spin effect on the adsorption energy.

Table S1. EXAFS fitting parameters at the Fe and Co K-edge various samples（S_0^2=0.67, 0.78）

Sample	Path	C.N.	R (Å)	$\sigma^2\times10^3$ (Å^2)	ΔE (eV)	R factor
Fe foil	Fe-Fe	8*	2.47±0.01	4.4±1.2	-1.5±1.5	0.002
	Fe-Fe	6*	2.84±0.01	5.3±2.2	-4.0±3.4	
Fe	Fe-S	5.5±0.3	2.25±0.01	4.6±4.3	0.1±0.6	0.002
Co foil	Co-Co	12*	2.50±0.01	6.5±0.1	7.4±0.3	0.001
Co	Co-S	5.3±0.6	2.25±0.01	6.0±1.2	-0.7±1.5	0.006

[a]*C.N.*: coordination numbers; [b]*R*: bond distance; [c]σ^2: Debye-Waller factors; [d] Δ*E*: the inner potential correction. *R* factor: goodness of fit. * the experimental EXAFS fit of metal foil by fixing CN as the known crystallographic value.

Table S2. Spin-state analysis of Fe single atoms. Room temperature ^{57}Fe Mössbauer parameters for ^{57}Fe enriched CFS-ACs/CNT

Fe species	IS (mm/s)	QS (mm/s)	Area	Line width (mm/s)
CFS-ACs/CNT	0.3052	0.5482	18970	0.1955

Table S3. Atomic distribution of CFS-ACs/CNT before and after OER stability

Elements	Atomic% after OER stability	Atomic% before OER stability
C	76.66	78.31
Co	0.77	1.31
Fe	0.64	1.28
S	14.8	19.01
O	7.13	0.09

Table S4. OER performance of transition metal-based catalysts in 1.0 M KOH

Catalysts	j (mA/cm^2)	Overpotentials (mV)	Stability (h)	Ref.
Fe_3C-Co/NC	10	370	16	[4]
Co_9S_8@MoS_2/CNFs	10	430	12	[5]
Mn-CoP	10	288	24	[6]
FePc-GO	10	320	16	[7]
Co-POP	10	340	10	[8]
La(CrMnFeCo_2Ni)O_3	10	325	50	[9]
CoMM	10	351	300	[10]
Co-N-C	10	321	16	[11]
CoMoP/CoNWs	20	270	40	[12]
NiFeP/MXene	10	286	12	[13]
FeCoWMo_4	10	276	50	[14]

Table S5. The summary for medium spin state during different fields.

Samples	Valence	Spin state	Applications	Regulatory approach	Key intermediates adsorption	Ref.
Fe,Mn-N-C	Fe (III)	M.S. ($t_{2g}^4 e_g^1$)	Oxygen reduction reaction (ORR)	Mn-N activates the FeIII sites by electronic modulation	Antibonding π-orbital of oxygen	[15]
o-MQFe	Fe (III)	M.S. ($t_{2g}^4 e_g^1$)	ORR	Axial Fe-O-Ti ligand regulation	Optimize O_2 adsorption by FeN_3O	[16]
Fe_{SA}-NSC	Fe (II)	M.S. ($t_{2g}^6 e_g^1$)	Nitrogen reduction reaction	Incorporation of S	Facilitating e_g electrons to penetrate the antibonding π-orbital of nitrogen	[17]
Fe-N-C/ Pd_{NC}	Fe (II)	M.S. ($t_{2g}^5 e_g^1$)	ORR	Pd_{NC}-induced	Activating O–O bond through the side-on overlapping	[18]
Fe-CoOOH	Co (III)	M.S. ($t_{2g}^5 e_g^1$)	Oxygen evolution reaction (OER)	Orbital occupancy of e_g	Possessing slightly strong adsorption energy	[19]

References

1. G. Sun, P. Sautet, Metastable Structures in Cluster Catalysis from First-Principles: Structural Ensemble in Reaction Conditions and Metastability Triggered Reactivity. *J. Am. Chem. Soc.* **140**, 2812-2820 (2018).
2. W. Peng *et al.*, Deciphering the Dynamic Structure Evolution of Fe- and Ni-Codoped CoS_2 for Enhanced Water Oxidation. *ACS Catal.* **12**, 3743-3751 (2022).
3. K. Hedenstedt, J. Bäckström, E. Ahlberg, In-Situ Raman Spectroscopy of α- and γ-FeOOH during Cathodic Load. *J. Electrochem. Soc.* **164**, H621 (2017).
4. C. C. Yang, S. F. Zai, Y. T. Zhou, L. Du, Q. Jiang, Fe_3C-Co Nanoparticles Encapsulated in a Hierarchical Structure of N-Doped Carbon as a Multifunctional Electrocatalyst for ORR, OER, and HER. *Adv. Funct. Mater.* **29**, 1901949 (2019).
5. Y. Yang *et al.*, Hierarchical Nanoassembly of $MoS_2/Co_9S_8/Ni_3S_2/Ni$ as a Highly Efficient Electrocatalyst for Overall Water Splitting in a Wide pH Range. *J. Am. Chem. Soc.* **141**, 10417-10430 (2019).
6. Y. Liu *et al.*, Porous Mn-doped cobalt phosphide nanosheets as highly active electrocatalysts for oxygen evolution reaction. *Chem. Eng. J.* **425**, 131642 (2021).
7. W. Wan *et al.*, Bifunctional Single Atom Electrocatalysts: Coordination–Performance Correlations and Reaction Pathways. *ACS Nano* **14**, 13279-13293 (2020).
8. H. Lei *et al.*, Metal-Corrole-Based Porous Organic Polymers for Electrocatalytic Oxygen Reduction and Evolution Reactions. *Angew. Chem. Int. Ed.* **61**, e202201104 (2022).
9. T. X. Nguyen, Y.-C. Liao, C.-C. Lin, Y.-H. Su, J.-M. Ting, Advanced High Entropy Perovskite Oxide Electrocatalyst for Oxygen Evolution Reaction. *Adv. Funct. Mater.* **31**, 2101632 (2021).
10. P. Kumar *et al.*, High-Density Cobalt Single-Atom Catalysts for Enhanced Oxygen Evolution Reaction. *J. Am. Chem. Soc.* **145**, 8052-8063 (2023).
11. D. Lyu *et al.*, Highly Efficient Multifunctional Co–N–C Electrocatalysts with Synergistic Effects of Co–N Moieties and Co Metallic Nanoparticles Encapsulated in a N-Doped Carbon Matrix for Water-Splitting and Oxygen Redox Reactions. *ACS Appl. Mater. & Inter.* **11**, 39809-39819 (2019).
12. V. H. Hoa *et al.*, Molybdenum and Phosphorous Dual Doping in Cobalt Monolayer Interfacial Assembled Cobalt Nanowires for Efficient Overall Water Splitting. *Adv. Funct. Mater.* **30**, 2002533 (2020).
13. J. Chen *et al.*, Vertically-interlaced NiFeP/MXene electrocatalyst with tunable electronic structure for high-efficiency oxygen evolution reaction. *Sci. Bull.* **66**, 1063-1072 (2021).
14. J. Zhang *et al.*, Novel monoclinic ABO_4 oxide with single-crystal structure as next generation electrocatalyst for oxygen evolution reaction. *Chem. Eng. J.* **420**, 130492 (2021).
15. G. Yang *et al.*, Regulating Fe-spin state by atomically dispersed Mn-N in Fe-N-C catalysts with high oxygen reduction activity. *Nat. Commun.* **12**, 1734-1744 (2021).
16. Y. Liu *et al.*, Tuning the Spin State of the Iron Center by Bridge-Bonded Fe-O-Ti Ligands for Enhanced Oxygen Reduction. *Angew. Chem. Int. Ed.* **61**, e202117617 (2022).
17. Y. Li *et al.*, Local spin-state tuning of iron single-atom electrocatalyst by S-coordinated doping for kinetics-boosted ammonia synthesis. *Adv. Mater.* **34**, 2202240 (2022).

18. X. Wei *et al.*, Tuning the spin state of Fe single atoms by Pd nanoclusters enables robust oxygen reduction with dissociative pathway. *Chem* **9**, 181-197 (2023).
19. W. H. Lee *et al.*, Electrode reconstruction strategy for oxygen evolution reaction: maintaining Fe-CoOOH phase with intermediate-spin state during electrolysis. *Nat. Commun.* **13**, 605 (2022).
